# Supplementary material for: Pediatric growth hormone treatment in Italy: A systematic review of epidemiology, quality of life, treatment adherence, and economic impact
Source: PLoS One. 2022 Feb 25;17(2):e0264403. doi: 10.1371/journal.pone.0264403 (PMC8880399; doi:10.1371/journal.pone.0264403)
Supplement: S2 Table — (DOCX) [file pone.0264403.s003.docx]

**S2 Table. List of excluded studies and reasons for exclusion.**

| **Reference** | **Reason for exclusion** |
| --- | --- |
| 1. Antoniazzi, F., et al. (2010). "GH in combination with bisphosphonate treatment in osteogenesis imperfecta." Eur J Endocrinol 163(3): 479-487. | Study objective |
| 1. Cambiaso, P., et al. (2017). "Growth hormone excess in children with neurofibromatosis type-1 and optic glioma." American Journal of Medical Genetics, Part A 173(9): 2353-2358. | Study objective |
| 1. Cappa, M., et al. (2016). "Efficacy and safety of growth hormone treatment in children with short stature: the Italian cohort of the GeNeSIS clinical study." J Endocrinol Invest 39(6): 667-677. | Not an epidemiological study |
| 1. Child, C. J., et al. (2019). "Safety Outcomes During Pediatric GH Therapy: Final Results From the Prospective GeNeSIS Observational Program." J Clin Endocrinol Metab 104(2): 379-389. | Not an epidemiological study |
| 1. Gallo, P., et al. (2016). "SGA Children in Pediatric Primary Care: What Is the Best Choice, Large or Small? A 10-Year Prospective Longitudinal Study." Glob Pediatr Health 3: 2333794x16659993. | Study objective |
| 1. Ghirri, P. and A. Bartoli (2010). "The small for gestational age (SGA) newborn." Rivista Italiana di Medicina dell'Adolescenza 8(3 SUPPL. 1): 3-7. | Not an epidemiological study |
| 1. Giavoli, C., et al. (2012). "GH replacement improves quality of life and metabolic parameters in cured acromegalic patients with growth hormone deficiency." J Clin Endocrinol Metab 97(11): 3983-8. | Adult patients |
| 1. Grugni, G., et al. (2016). "Growth hormone therapy for Prader-willi syndrome: challenges and solutions." Ther Clin Risk Manag 12: 873-881. | Not an epidemiological study |
| 1. Jommi, C. (2013). "Economic evaluation of growth hormone treatment in children and adolescents." Rivista Italiana di Medicina dell'Adolescenza 11(3 SUPPL. 1): 32-35. | Full-text not available |
| 1. Meazza, C., et al. (2013). "3-M syndrome associated with growth hormone deficiency: 18 year follow-up of a patient." Ital J Pediatr 39: 21. | Study objective |
| 1. Meazza, C., et al. (2013). "Development of antibodies against growth hormone (GH) during rhGH therapy in a girl with idiopathic GH deficiency: a case report." J Pediatr Endocrinol Metab 26(7-8): 785-788. | Study objective |
| 1. Menke, L. A., et al. (2010). "The effect of the weak androgen oxandrolone on psychological and behavioral characteristics in growth hormone-treated girls with Turner syndrome." Hormones and Behavior 57(3): 297-305. | Study objective |
| 1. Muscogiuri, G., et al. (2019). "Prader- Willi syndrome: An uptodate on endocrine and metabolic complications." Rev Endocr Metab Disord 20(2): 239-250. | Not an epidemiological study |
| 1. Nobile, S., et al. (2017). "Neonatal outcome of small for gestational age preterm infants." Eur J Pediatr 176(8): 1083-1088. | Not an epidemiological study |
| 1. Quitmann, J., et al. (2017). "Validation of the Italian Quality of Life in Short Stature Youth (QoLISSY) questionnaire." J Endocrinol Invest (2017) 40:1077–1084. | Did not report quality of life outcomes. This study was described in the Discussion section. |
| 1. Quitmann, J., et al. (2019). "First-year predictors of health-related quality of life changes in short-statured children treated with human growth hormone." J Endocrinol Invest 42(9): 1067-1076. | Study conducted in countries other than Italy |
| 1. Rapaport, R., et al. (2013). "Validation and ease of use of a new pen device for self-administration of recombinant human growth hormone: Results from a two-center usability study." Medical Devices: Evidence and Research 6(1): 141-146. | Study objective |
| 1. Sävendahl, L., et al. (2020). "Long-term mortality after childhood growth hormone treatment: the SAGhE cohort study." Lancet Diabetes Endocrinol 8(8): 683-692. | Not an epidemiological study |
| 1. Sbardella, E., et al. (2019). "ENDOCRINOLOGY AND ADOLESCENCE: Dealing with transition in young patients with pituitary disorders." Eur J Endocrinol 181(4): R155-r171. | Study objective |
| 1. Spada, E., et al. (2018). "Effect of maternal age, height, BMI and ethnicity on birth weight: an Italian multicenter study." Journal of Perinatal Medicine 46(9): 1016-1021. | Not an epidemiological study |
| 1. Swerdlow, A. J., et al. (2015). "Description of the SAGhE cohort: A large european study of mortality and cancer incidence risks after childhood treatment with recombinant growth hormone." Hormone Research in Paediatrics 84(3): 172-183. | Not an epidemiological study |
| 1. Swerdlow, A. J., et al. (2017). "Cancer Risks in Patients Treated With Growth Hormone in Childhood: The SAGhE European Cohort Study." J Clin Endocrinol Metab 102(5): 1661-1672. | Not an epidemiological study |
| 1. Swerdlow, A. J., et al. (2018). "Risk of Meningioma in European Patients Treated with Growth Hormone in Childhood: Results from the SAGhE Cohort." Journal of Clinical Endocrinology and Metabolism 104(3): 658-664. | Not an epidemiological study |
| 1. van Huis, M., et al. (2016). "Considerable variations in growth hormone policy and prescription in paediatric end-stage renal disease across European countries-a report from the ESPN/ERA-EDTA registry." Nephrol Dial Transplant 31(4): 609-619. | Study objective |
| 1. Zeitlin, J., et al. (2017). "Variation in term birthweight across European countries affects the prevalence of small for gestational age among very preterm infants." Acta Paediatr 106(9): 1447-1455. | Study objective |
